# Digital empowerment of dance movement therapy: innovative applications and evidence-based evaluation in mental health interventions

**DOI:** 10.3389/fpsyg.2026.1935660

**Published:** 2026-09-28

**Authors:** Qilin Zhang, Jianjun Wu, Yifan Fang, Dong Yang, Yu Cheng

**Affiliations:** 1 College of Physical Education, Hunan Normal University, Changsha, China; 2 School of Physical Education and Health Science, Guangxi Minzu University, Nanning, China; 3 Sports Department, Zhejiang Automotive Vocational and Technical College, Taizhou, China; 4 College of Physical Education, Chongqing University, Chongqing, China

**Keywords:** cross-cultural local adaptation, dance movement therapy, digital health, evidence-based practice, integrated sports and medicine, multi-modal quantitative assessment

## Abstract

**Background and objectives:**

Mental health disorders are a severe global public health crisis. Dance movement therapy (DMT) offers unique benefits, but traditional DMT is limited by subjective evaluation, non-standardized protocols, and restricted access. Digital technologies such as virtual reality, artificial intelligence, and wearable sensors may address these limitations; however, a structural gap remains between technological application and rigorous clinical evidence.

**Methods:**

This narrative review synthesizes literature from PubMed, Web of Science, Scopus, and CNKI. Sources used for population-specific synthesis were coded by population, intervention modality, outcome measures, study design, and relationship to the review question (direct digital-DMT; digital but non-DMT; conventional DMT/dance; mechanistic/contextual). Claims that extend beyond primary digital-DMT evidence are flagged as interpretive contributions or as findings from adjacent analogue sources.

**Results:**

Digital technologies can make DMT more multi-modal and quantitative, but direct trial evidence for digital DMT remains sparse and heterogeneous. Preliminary support for anxiety-related outcomes comes mainly from one small randomized trial of digital dance-art therapy and from mixed-methods online dance studies. Most population-specific estimates in this review derive from conventional (non-digital) DMT/dance syntheses or from digital but non-DMT analogues (for example, VR exposure therapy or home-based exergaming) and should not be read as direct digital-DMT effects. Conventional long-term dance/DMT programmes show modest cognitive improvements in older adults with mild cognitive impairment, while evidence for Alzheimer’s disease remains inadequate. In China, Western theoretical frameworks and cultural constraints also limit localization.

**Conclusion:**

Digital DMT requires deep interdisciplinary integration rather than simple technological combination. This review proposes a three-level localization framework for China and calls for large-sample randomized controlled trials, stronger data-privacy protections, and narrowed digital divides.

## 1 Introduction

Mental health crises constitute one of the most urgent global public health challenges of the 21st century. According to 2022 World Health Organization (WHO) statistics, approximately one billion individuals globally live with mental disorders, and depressive and anxiety disorders account for 13% of the total global disease burden. Following the COVID-19 outbreak, the worldwide incidence of depression and anxiety rose by 25%, while low- and middle-income countries bear a 75% unmet gap in mental healthcare provision (Millman et al., 2021). Conventional mental health interventions are inherently constrained in service accessibility, personalised treatment design and objective efficacy evaluation, necessitating innovative non-pharmacological intervention alternatives. As a psychotherapy modality relying primarily on bodily movement, Dance Movement Therapy (DMT) facilitates emotional integration, stress alleviation and self-awareness enhancement via embodied, nonverbal physical expression. Systematic reviews confirm consistent dance-based interventions effectively alleviate depressive, anxious and psychological distress symptoms across adult populations regardless of dance genres (standardised mean difference, SMD = −0.52, 95% confidence interval, CI: −0.73 to −0.31) (Moratelli et al., 2023). Nonetheless, traditional DMT is hindered by three fundamental evidence-based limitations: therapeutic outcomes predominantly depend on subjective observation and self-reported questionnaires lacking objective quantitative metrics; intervention protocols remain highly individualised with minimal cross-study standardisation; and in-person delivery models restrict large-scale population outreach.

Digital technologies—extended reality (XR), AI/ML, wearable biosensing and big data—are reshaping this landscape by enabling objective, quantifiable and personalized intervention (Huang et al., 2023), and sports science offers complementary methodological grounding for evidence-based program design. Yet this technological capability has outpaced the clinical evidence base: rigorously designed RCTs and standardized intervention classifications remain scarce (Minetti et al., 2025), and unresolved risks around the digital divide, data privacy and the changing therapist role compound a structural tension between rapid technological development and lagging evidence translation. Prior reviews have generally examined either the technological components of digital DMT or its psychotherapeutic outcomes in isolation (Koch et al., 2019); few integrate sports-science-based quantitative evaluation, population-specific evidence classification and cross-cultural adaptation within a single framework. This integrative, three-strand synthesis is the central contribution and novelty of the present review.

China offers a particularly urgent and instructive case for closing this gap. It carries one of the largest absolute burdens of mental disorder worldwide alongside sharply uneven urban–rural resource distribution (Huang et al., 2019; GBD 2019 Mental Disorders Collaborators, 2022; Liu et al., 2011), magnifying the public-health value of scalable digital interventions; national policy has recently prioritized digital and smart-health infrastructure (General Office of the State Council of the People's Republic of China, 2018), opening a narrow window in which evidence-informed design can still shape large-scale implementation; and the theoretical architecture of existing digital DMT platforms, developed almost entirely within individualistic, externalization-oriented Western contexts (Koch and Fischman, 2011), is unlikely to transfer successfully into a cultural environment shaped by collectivist values, affective restraint and psychiatric stigma without deliberate localization. Because this mismatch remains unaddressed in the literature, the Chinese case is both theoretically instructive and practically time-sensitive.

Against this background, this review aims to: (1) clarify the theoretical basis of digital DMT and its evolving intervention modes; (2) analyze intervention effectiveness and evidence classification across population groups; (3) formulate a situated localization path for China’s social and cultural context; and (4) identify key methodological obstacles and priority research directions in this field.

## 2 Methods

This review follows a narrative rather than a systematic design, reflecting the breadth and conceptual heterogeneity of literature spanning digital-health engineering, sports biomechanics, clinical psychotherapy and cross-cultural theory, which does not lend itself to a single homogeneous meta-analytic protocol. Literature was retrieved from major biomedical, psychological and sports-science databases (PubMed, Web of Science, Scopus and CNKI) using structured keyword combinations organized around six thematic clusters: dance/movement therapy (e.g., “dance movement therapy,” “dance therapy”), digital health (e.g., “digital health,” “telehealth”), extended reality (e.g., “virtual reality,” “augmented reality,” “extended reality”), wearable biosensing (e.g., “wearable sensor,” “heart rate variability,” “motion capture”), embodied cognition, and cross-cultural mental health, combined within each cluster using OR and across clusters using AND (e.g., (“dance movement therapy” OR “dance therapy”) AND (“virtual reality” OR “extended reality” OR “wearable sensor”)). Searches were restricted to peer-reviewed sources published within the past 5 years (2021–2026), unless a source held foundational theoretical significance for embodied cognition or cross-cultural adaptation theory, in which case earlier landmark publications were retained regardless of date; sources were further restricted to English- and Chinese-language publications, consistent with the two linguistic and cultural contexts (Western clinical literature and Chinese localization literature) this review compares in Section 5.

An initial search across the four databases yielded approximately 6,500 records. After removing duplicates and screening titles, abstracts and full texts against the criteria below, 24 sources were retained for formal coding in the study-level narrative evidence map (Table 1). Additional theoretical, policy and cultural sources cited in Sections 1, 5 and 6 were used for contextual interpretation and are not part of the coded map. This screening ratio is consistent with the broad, high-recall search adopted here, which prioritized topical coverage across six clusters over search precision. Inclusion required that a source be (a) an empirical intervention, a systematic synthesis, or a theoretical/conceptual contribution relevant to digital DMT or to an explicitly labelled adjacent analogue; (b) for empirical studies, a specified population, intervention modality and outcome measure; and (c) accessible in full text. Non-peer-reviewed commentary and sources lacking full text were excluded. Conference abstracts without a retrievable full paper were excluded; full-text conference proceedings that otherwise met coding criteria were retained and flagged by study type (Al-Mfarej et al., 2023). Adjacent analogue sources (conventional DMT/dance, digital non-DMT interventions, and mechanistic papers) were retained only where they informed baselines, delivery analogues or candidate mechanisms; they were not used as direct evidence that digital DMT is effective.

**Table 1:** Evidence matrix of digital DMT across target populations (narrative evidence map, not a formal GRADE assessment; 24 formally coded sources).

Citation	Study type	Target population	Sample size	Intervention course/duration	Intervention description	Outcome measures	Main findings	Adverse events/Safety observations	Evidence class	Key limitations
Children and adolescents										
Tao et al. (2022)	Systematic review	Children and adolescents	NR	NR	Structured dance-based physical activity interventions across included primary studies	PTSD-related symptoms, psychological well-being scales, motor function	Dance-related interventions deliver broad physiological-psychological benefits for clinical and non-clinical youth populations	NR	C	High heterogeneity for intervention parameters across primary studies; limited long-term follow-up
Li et al. (2025)	Systematic review	Children and adolescents with PTSD	NR	NR	Digital arts-therapy interventions	Validated PTSD rating scales	Digital art therapy yields preliminary symptom-relieving effects for minors with PTSD	NR	C	Component primary studies mostly small sample; heterogeneous digital-art-therapy modalities
Prosperini et al. (2026)	RCT	Adults with multiple sclerosis	NR	NR	Home-based virtual exergame cognitive-motor intervention	Cognitive function, spatial attention, visual-motor integration metrics	Action-based virtual interventions produce incidental cognitive benefits via visual-movement integration	NR	B	Study population is adults with multiple sclerosis; findings cannot be directly generalized to child-adolescent PTSD/ADHD
Kim et al. (2022)	Review	Children and adolescents with functional neurological disorder (FND)	NR	NR	Games, music and dance integrated into physical rehabilitation as a core component of multi-modal FND management	Functional motor-symptom severity; rehabilitation engagement indicators	Multi-modal intervention combining dance/movement elements is identified as a core component of pediatric FND rehabilitation	NR	D	Narrative/clinical review synthesis rather than primary controlled-trial evidence
Finn et al. (2023)	Mixed-methods study	Young people aged 16–24 living with anxiety	NR	NR	Online group dance sessions as social and emotional support	Anxiety-related well-being indicators; qualitative social-connection themes	Online group dance provides a supportive “social cure” and a non-verbal emotional-expression pathway for anxious young people	NR	B	Mixed-methods design with a qualitative component; limited generalizability beyond self-selected online-dance participants
Al-Mfarej et al. (2023)	Pilot/feasibility study	Children with muscular dystrophy	NR	NR	Clinician-guided VR exergame combining breathing and relaxation exercises with tailored sensory input	Emotional-regulation and relaxation indicators	Tailored sensory input within VR exergames supports precise emotional adjustment in this clinical pediatric population	NR	B	Conference-proceedings report rather than peer-reviewed journal trial; single clinical pediatric subgroup
Richlan et al. (2023)	Narrative review	General/sport-training populations (applied to ADHD-relevant intervention design)	NR	NR	VR-based hierarchical task progression with rhythmic active-strengthening training	Motivation indicators; prefrontal-cortex-linked attentional/executive-function proxies	Structured VR training with graded task difficulty and rhythmic activity may enhance motivation and executive/attentional function relevant to ADHD-focused DMT design	NR	B	Narrative review of general VR sports-training literature, not DMT- or ADHD-specific primary evidence
Older adults (MCI/Dementia)										
Huang et al. (2023)	Systematic review & meta-analysis	Adults ≥55 years with mild cognitive impairment	NR	NR	Dance-therapy interventions; benefits are stronger for interventions ≥3 months duration	Cognitive assessment scales, mental-health symptom inventories	Dance therapy improves multi-domain cognitive performance and alleviates depressive symptoms in older-adult MCI participants	NR	C	Choreography protocols heterogeneous across trials; few large-sample RCTs
Ho et al. (2020)	RCT	Older adults with mild dementia	NR	12 weeks	12-week group-based DMT vs. waiting-list control	Depressive-symptom scales, activities-of-daily-living scales, cortisol rhythm metrics	Modest reduction of mild depressive symptoms; improved daily-living capacity; optimized daytime cortisol rhythm; cognitive-neuropsychiatric improvements remain inconclusive	NR	C	Single small-sample RCT; insufficient for robust dementia clinical recommendations
Murillo-Garcia et al. (2021)	Systematic review & meta-analysis	Middle-aged and older adults	NR	NR	Dance-therapy interventions	Dual-task performance, balance, motor-cognitive synchronization indicators	Dance interventions significantly improve dual-task performance and motor-cognitive coordination in older adults	NR	C	Intervention protocols heterogeneous; few trials focus on dementia subgroups
Wu et al. (2022)	Systematic review	Older adults: Parkinson’s disease, MCI, Alzheimer’s disease	NR	NR	Digital-DMT and dance-movement interventions	Motor-function and cognitive-function assessment scales	Sustained benefits for PD motor disorder and MCI-related cognitive decline; evidence for Alzheimer’s disease remains limited	NR	C	Small sample sizes in primary studies; variable testing methods; lack of standardized protocols
Chronic disease and special clinical groups (Anxiety, PD, Breast Cancer, Eating-disorder/Early-psychosis populations)										
Kothgassner et al. (2023)	Narrative review	People with anxiety-spectrum disorders	NR	NR	Virtual-reality exposure therapy (VRET)	Anxiety-symptom scales; odds-ratio effect estimation	VR-based embodied exposure yields robust clinical effects for anxiety-related disorders	Cybersickness risk noted for immersive-VR applications	B	Evidence originates from VRET research; only partial transferability to digital-DMT
Vignapiano et al. (2026)	Primary observational study	Early-psychosis and eating-disorder clinical samples	NR	NR	Dance-movement therapy compared with art therapy	Somatic-tension indicators, emotion-regulation clinical scales	DMT mainly alleviates somatic tension among eating-disorder patients; evidence for emotion-regulation effects in psychosis remains limited	NR	C	Single-study evidence base; relatively small sample
Fatkulina et al. (2021)	Systematic review	Breast-cancer survivors	NR	NR	Dance-movement therapy interventions	Psychological well-being, disease-related symptom indicators	Positive psychological well-being effects for breast-cancer survivors receiving DMT	NR	C	Limited eligible RCT quantity
Abu-Odah et al. (2024)	Systematic review & meta-analysis	Cancer patient populations	NR	NR	Dance-movement therapy interventions	Quality-of-life outcome metrics	Pooled quality-of-life outcomes across two RCTs non-significant (SMD − 0.09, 95%CI − 0.21–0.40)	NR	C	Few RCTs; substantial between-study heterogeneity
Raghupathy et al. (2022)	RCT	Children with Down syndrome	NR	NR	Traditional Indian dance training vs. conventional neuromuscular rehabilitation	Locomotor proficiency, static-balance assessment	Traditional Indian dance training improves motor-skill and balance outcomes superior to conventional rehabilitation	NR	C	Sample restricted to children with Down syndrome; limited generalizability
Millman et al. (2021)	Systematic review	Adults with mental-health conditions (neurocognitive focus)	NR	NR	Dance movement therapy examined through a neurocognitive lens (movement–brain-activity coupling)	Neurocognitive and mental-health outcome measures across included studies	DMT’s therapeutic efficacy is linked to bidirectional coupling between somatic movement and brain activity, providing neurocognitive grounding for embodied-cognition-based intervention design	NR	D	Heterogeneous primary studies; mechanistic evidence base still developing, limiting firm clinical conclusions
Occupational sub-healthy working-age populations										
Bi et al. (2026)	Meta-analysis	Broad adult population	NR	NR	Moderate-to-low-intensity continuous training vs. high-intensity interval training	Cardiovascular-physiological indicators (systolic blood pressure, autonomic-related metrics)	Moderate-low-intensity continuous training avoids pathological-stress-related physiological responses and improves autonomic-related physiological indices; original meta-analysis does NOT directly assess resting-state autonomic homeostasis	NR	D	Aggregates general exercise literature; it is not a DMT-specific trial
Xu et al. (2024)	Conceptual-empirical paper	Adults accessing psychological-assistance hotlines	NR	NR	DMT-adapted working model for telephone-based psychological hotline service	Qualitative process evaluation; service-delivery feasibility indicators	Feasible DMT working model for psychological hotline-assistance settings	NR	A (conceptual)	No physiological-stress-reduction outcome data; this paper does NOT report indigenous Korean DMT
Qiu et al. (2025)	RCT	Dancers with none-to-mild anxiety	*N* = 48	3 months (twice-weekly)	Smart-fitness-mirror-guided digital dance-art therapy vs. traditional studio dance	Anxiety-related scales; Positive and Negative Affect Schedule (PANAS)	Digital dance-art therapy reduced anxiety-related scores relative to traditional studio dance; positive affect improved more clearly than negative affect, which remained mixed	NR	A	Small sample of dancers rather than a clinical anxiety-disorder cohort; PANAS is not a dedicated anxiety diagnostic scale
Radanliev (2024)	Conceptual	Occupational sub-health populations (proposed)	NR	NR	XR platform (MR/AR/VR head-mounted displays and tactile wearables) proposed for occupational stress-relief content	None (design proposal)	Describes a technically feasible XR platform for occupational stress relief; ‘precision’ is presented as a design goal rather than a demonstrated outcome	NR	A (conceptual)	No empirical outcome data; conceptual/design proposal only
Radanliev (2025)	Conceptual	Anxiety-spectrum populations (proposed)	NR	NR	Conceptual XR-DMT programme integrating wearable biosensing and AI-driven adjustment, following a structured therapeutic frame (mirroring, embodied cognition, rhythm synchronization)	Proposed HRV/EDA and anxiety indicators (not yet measured)	Proposes that structured XR-DMT programmes can plausibly yield anxiety and HRV/EDA changes; presented explicitly as design hypotheses	NR	A (conceptual)	No empirical trial data; hypotheses pending adequately powered testing
Moratelli et al. (2023)	Systematic review	Adults (general, working-age)	NR	NR	Dance-based interventions across various dance genres	Depressive, anxiety and psychological-distress symptom scales	Dance interventions significantly reduce depressive, anxious and psychological-distress symptoms in adults (SMD = −0.52, 95% CI: −0.73 to −0.31), regardless of dance genre	NR	C	Primarily analogue (non-digital) dance interventions; digital/XR-specific evidence not separately reported
Aguilera et al. (2024)	RCT	Adults with diabetes and depression	NR	24 weeks	Reinforcement-learning-driven adaptive digital notification intervention promoting physical activity	Daily step counts; depression-related indicators	Reinforcement-learning-based adaptive digital intervention achieved continuous daily-step growth over 24 weeks, offering a methodological precedent for digital optimization of DMT-style interventions	NR	B	Digital step-count intervention, not a DMT-specific trial; population defined by diabetes/depression comorbidity rather than occupational sub-health

Table maps the 24 formally coded sources as a study-level narrative evidence map; it is not a GRADE assessment. Evidence class classifies each source’s relationship to the review question: A = direct digital-DMT; B = digital, non-DMT; C = conventional DMT/dance; D = mechanistic/contextual. Sample size, duration, comparator and adverse-event cells report information extracted from the source; NR = not reported in that source. For systematic reviews, k and pooled N are given when the review reports them; duration is listed as variable when protocols differ across included trials. Class B–D findings should not be read as direct digital-DMT effects. Only Figure 1 is included in this submission.

Each of the 24 Table 1 sources was independently coded by two reviewers against the full text for population, intervention modality, outcome measures, study design, sample size, duration, comparator and adverse-event reporting, with discrepancies resolved by discussion. Every source was assigned one mutually exclusive evidence class relative to the review question (digital DMT for mental health): (A) direct digital-DMT evidence (technology-mediated DMT or digital dance therapy framed as therapeutic dance/movement); (B) digital but non-DMT evidence (digital/VR/exergame or digital-health interventions without a DMT protocol); (C) conventional DMT/dance evidence (in-person DMT or dance without digital delivery); and (D) mechanistic/contextual evidence (exercise-physiology, neurocognitive or theoretical sources used to interpret mechanisms). This class structure, rather than study-level GRADE-informed certainty labels, is what allows the review to distinguish, for example, conventional dance/DMT findings in mild cognitive impairment from the much thinner direct digital-DMT corpus, and to avoid treating VR exposure therapy or step-count messaging trials as if they were digital DMT. Where sources disagreed, the disagreement is reported and the conclusion is restricted to the appropriate evidence class rather than being resolved by weighting an individual paper as ‘higher certainty’. GRADE is not applied at the individual-study level in this narrative map, because GRADE concerns certainty in a body of evidence for a specified outcome.

Claims that extend beyond what Class A sources directly support—including cultural-adaptation proposals in Section 5 and any inference from Class B–D analogues to digital DMT—are explicitly flagged as interpretive or conceptual contributions, or as adjacent-analogue findings, rather than as established digital-DMT effects, and are cross-checked against independent sources where possible. This distinction is maintained throughout Sections 3 through 6.

## 3 Theoretical foundations and paradigm shifts

### 3.1 Embodied cognition and technologically mediated restructuring of embodied experience

The integration of digital technologies into dance movement therapy has not merely transposed conventional practices into virtual spaces, but has precipitated a paradigmatic shift in how embodiment is conceptualized, experienced (Favela, 2020), and therapeutically leveraged, thereby demanding theoretical grounding in frameworks that recognize cognition as fundamentally situated in bodily action. The theoretical cornerstone of the integration of digital technology with dance movement therapy (DMT) comes from the theory of embodied cognition. The theory regards cognitive processing as a dynamic experience rooted in body perception and body movement, emphasizing the inseparable interaction between mind, body, and the surrounding environment (Favela, 2020). This theoretical framework carries profound implications for therapeutic practice: if cognition is inherently grounded in bodily experience, then interventions that restructure somatic engagement hold the potential to reshape not only behavioral patterns but also deeper cognitive and affective processes. From a neurocognitive perspective, the therapeutic efficacy of DMT depends substantially on bidirectional coupling between somatic movement and brain activity (Millman et al., 2021), a mechanism that provides further empirical grounding for applying embodied cognition theory within psychological intervention.

Scholars working at the intersection of phenomenology and digital health have argued that traditional dance movement therapy, though powerful in its therapeutic application of movement and somatic awareness, has historically been constrained by the limitations of physical space and the necessity of face-to-face embodied interaction (Koch and Fischman, 2011). Within this literature, the emergence of immersive digital environments—most notably those constructed through virtual reality (VR) technology—has been characterized as a qualitative expansion of these therapeutic possibilities (Radanliev, 2024). Proponents of this view suggest that, by enabling participants to inhabit and express themselves through digitally mediated bodies, VR transcends the spatial and contextual boundaries of conventional therapeutic settings, thereby opening new modalities of somatic exploration that are simultaneously familiar and transformative (Koch and Fischman, 2011). Building on this literature, we interpret VR as not merely supplementing existing therapeutic frameworks but actively participating in the phenomenological restructuring of embodied experience itself; this claim is an interpretive, theory-building proposition of the present review rather than a directly measured empirical finding, and it is offered here as a conceptual bridge to the evidence reviewed in the remainder of this section.

This restructuring has particularly significant implications for the treatment of anxiety-spectrum disorders, where the relationship between body, environment, and perceived threat constitutes the very architecture of pathological experience. Virtual reality exposure therapy (VRET) operationalizes the principles of systematic desensitization within graded, precisely controllable virtual environments, allowing clinicians to calibrate the intensity and nature of exposure stimuli with a degree of precision unattainable in conventional *in vivo* conditions. The clinical efficacy of this approach has been substantiated by robust empirical evidence across multiple randomized controlled trials, yielding notably favorable outcomes (odds ratio OR = 0.36, 95% confidence interval: 0.23–0.57) (Kothgassner et al., 2023). These VRET findings are Class B (digital but non-DMT) analogue evidence: they support the proposition that technologically mediated restructuring of embodied experience can be therapeutically useful, but they are not direct evidence of digital DMT efficacy, and any transfer to DMT remains a hypothesis.

### 3.2 Paradigm innovation in DMT evaluation driven by quantitative technology integration

Nevertheless, practices of motion sensing in metaverse and extended-reality environments indicate that, while wearable devices can collect abundant somatic data, sensor noise and inter-individual somatic heterogeneity constrain the clinical interpretive validity of these physiological metrics, such that psychological intervention efficacy cannot be inferred directly from a single physiological parameter (Radanliev, 2024). Real-time synchronous monitoring of heart rate variability (HRV), skin electrical activity (EDA), electroencephalogram (EEG), and the dynamic association between them and physical movement and virtual environment feedback has built a closed-loop tracking system for physical and mental functions (Radanliev, 2025). From the perspective of exercise physiology, the resting heart rate variability of patients with anxiety disorders is significantly lower than that of the healthy control group (Cohen’s d = 0.61, 95% CI: 0.47–0.75), which is consistent with the hypothesis that HRV change may serve as a candidate physiological correlate of intervention response; however, a cross-sectional between-group difference does not by itself establish HRV improvement as a validated indicator of treatment efficacy, and this distinction should be read as an interpretive hypothesis rather than a confirmed finding (Cheng et al., 2022). The inclusion of standardized motor function assessment scales, such as the Berg balance scale, the stand-walking test (TUG) and the Fugl-Meyer motor function score, significantly improves the comparability of treatment outcomes between different studies.

### 3.3 Reconstruction of the therapeutic relationships based on human-computer collaboration in the digital age

Digital innovation reshapes the therapeutic relationship in traditional dance movement therapy (DMT) into a collaborative framework composed of therapists, intelligent technology systems and visitors. A review of remote digital DMT delivery indicates that automated feedback can expand service accessibility, yet it also changes how therapeutic alliances are formed, and subtle emotional signals expressed through the body are prone to being filtered out by algorithmic mediation. Conceptual work on XR/metaverse DMT describes platforms that can analyse remote participants’ kinematic characteristics and provide audio and video feedback on movement deviations, while adjusting task difficulty according to individual bodily adaptability (Radanliev, 2024). This capacity for continuous, algorithmically mediated feedback clearly improves consistency and reach relative to intermittent in-person supervision; it also carries a countervailing risk, however. When biomechanical correction is delivered primarily through automated audio-visual cues rather than an attentive human observer, subtle affective signals—hesitation, dissociation, or emotional overwhelm expressed through posture rather than overt movement error—may go undetected, potentially narrowing the clinical sensitivity that expert therapists traditionally provide.

The collaborative model extends further into whole-program personalization: by combining artificial intelligence and machine learning algorithms with wearable biosensing data to achieve on-demand adjustment of the intervention program, this marks an important advance in accurate mental health service delivery (Radanliev, 2024) While this personalization pipeline promises more precisely calibrated interventions at scale, it also concentrates clinical judgment inside proprietary algorithms whose decision logic is frequently opaque to both therapist and client, which raises accountability and interpretability concerns should an automated adjustment prove clinically inappropriate for a given individual.

Correspondingly, treatment practitioners gradually transform into designers of intervention programs, technical supervisors and providers of remote emotional support, and the implementation of decentralized clinical trials (DCT) confirms that this collaborative model measurably reduces the travel burden on service recipients. This role transformation undeniably lowers geographic and logistical barriers to care; at the same time, it narrows the traditional scope of therapist practice toward technical supervision, which may, over time, erode the relational and improvisational skills that constitute much of DMT’s original therapeutic value, and it raises unresolved questions about whether client trust and disclosure are equivalently supported in a technology-mediated relationship as in an unmediated one. The central question this raises is therefore not simply whether technology can quantify or correct movement, but whether, and under what conditions, it preserves the core constructs that distinguish DMT as a psychotherapeutic modality from exercise training or movement correction – movement attunement and mirroring between therapist and client, kinesthetic empathy, symbolic and metaphorical expression through movement, psychological containment of affect, group process, trauma-informed practice, and the exercise of therapist clinical judgment in the moment. Digital systems that quantify kinematics without attending to these constructs may improve measurement precision while leaving the specifically therapeutic mechanism of DMT unaddressed.

## 4 Evidence synthesis of digital DMT: intervention efficacy, mechanistic pathways and population-specific adaptation

### 4.1 Children and adolescents: gamified digital interventions for physical and psychological development

Evidence in this age group is mixed in class, and Class A digital-DMT trials are essentially absent. Direct technology-mediated dance evidence is limited to small mixed-methods online dance work (Finn et al., 2023; Class B). Most remaining citations are conventional dance or arts-therapy syntheses (Class C) or digital non-DMT analogues (Class B) and are summarized as such; they are not treated as digital-DMT effects. For children and adolescents, digital and arts-based programmes often adopt game-like interactive frameworks to promote emotional recognition, social-ability cultivation and correction of clinical disorders. A systematic review of arts therapies (not digital DMT) found that for minors with post-traumatic stress disorder (PTSD), arts therapy has shown preliminary symptom-relieving effects, though modality-specific efficacy remains inconclusive (Li et al., 2025; Class C). As a creative form of physical activity, structured dance participation can bring a wide range of physiological and psychological benefits to adolescents with and without clinical conditions (Tao et al., 2022; Class C). Integrating games, music and dance into physical rehabilitation has been described as a component of multi-mode intervention for children’s functional neurological disorders (FND) (Kim et al., 2022; Class D). Online group dance can provide a non-verbal support path for young people living with anxiety (Finn et al., 2023; Class B). Tailor-made sensory input in a VR exergame has been reported as feasible in a very small muscular-dystrophy feasibility sample (Al-Mfarej et al., 2023; Class B); action-based virtual exergames produced incidental cognitive benefits in adults with multiple sclerosis, a finding that cannot be generalized to child-adolescent PTSD/ADHD or to DMT (Prosperini et al., 2026; Class B). Claims that somatosensory dance games constitute an evidence-based ADHD digital-DMT protocol exceed the present corpus: the cited VR sports-training narrative (Richlan et al., 2023; Class B) is an indirect design analogue only. Motion capture can quantify kinematic patterns for evaluation (Yan, 2020), and sports-training principles of gradual overload can guide dosing, as illustrated by an in-person Indian-dance RCT in children with Down syndrome (Raghupathy et al., 2022; Class C), which is neither DMT nor digital.

### 4.2 Elderly population: cognitive and physical outcomes of dance/DMT and implications for digital delivery

Direct digital-DMT evidence in older adults is currently lacking in Table 1; the findings below are predominantly Class C (conventional DMT/dance) unless marked otherwise, and they should not be restated as digital-DMT effects. Conventional DMT has been reported to improve quality of life, daily activity participation, and well-being among older adults. Adjacent aerobic-exercise evidence (Class D) indicates that long-term adherence to aerobic exercise is associated with better cardiopulmonary function (mean difference, MD = 11.36 mL/kg/min, 95% CI: 5.63–17.09, *p* < 0.01) and reduced body fat percentage (MD = −5.41, 95% CI: −7.65 to −3.17%, *p* < 0.01) (Leslie et al., 2023). Design principles proposed for older-adult DMT emphasize safety first, gradual intensity, and functional adaptation, while avoiding high-impact movements. In-person DMT trials in mild dementia further indicate that programmes must account for fall risk and functional limitation (Ho et al., 2020; Class C); for any future digitally delivered programme, device-use barriers and sensory-adaptation demands would additionally apply, or accessibility and safety will be compromised. A large body of empirical studies of conventional dance/DMT shows improvements in overall cognition, situational memory, executive function, attention control, and language ability in older adults, while alleviating depressive and neuropsychiatric symptoms. However, evidence for processing speed, visual–spatial ability, and quality of life is insufficient, and mechanisms remain incompletely specified (Huang et al., 2023; Class C). Intervention duration is a critical factor: programmes lasting more than 3 months yield significantly greater cognitive improvements than short-term interventions. Meta-analysis indicates that dance/DMT, as a non-pharmacological intervention, can enhance multidimensional cognitive abilities and alleviate depressive symptoms in elderly individuals with mild cognitive impairment (MCI); complex choreography that simultaneously mobilizes attention, memory, and executive functions is a candidate mechanism (Huang et al., 2023; Class C). Pairing simple rhythmic movements with complex memory tasks, or complex sequences with simpler cognitive tasks, is a design heuristic for avoiding excessive dual-task load in future digital protocols, not a finding already demonstrated for digital DMT. High-quality evidence for dementia remains limited: a 12-week group DMT versus waitlist trial (Ho et al., 2020; Class C) showed modest effects on mild depressive symptoms (d = 0.33–0.42, *p* < 0.05), improved daily activity ability (d = 0.40, *p* < 0.01), and optimized daytime cortisol rhythm (d = 0.30, *p* < 0.01), but neuropsychiatric and cognitive improvements remain insufficient for clear clinical recommendations.

Conventional dance-based programmes can bring both physical and cognitive benefits to older adults; this is Class C evidence. Included pilot and trial cycles range from 8 to 52 weeks, with 1 to 3 sessions a week lasting 40 to 90 min; meta-analysis shows that dance intervention significantly improves dual-task performance (effect size = −0.48, 95% CI: −0.88 to −0.08) and motor–cognitive coordination, including cognitively loaded TUG timing and walking speed during a concurrent cognitive task (Murillo-Garcia et al., 2021; Class C). In neurodegenerative disease, a systematic review of predominantly in-person dance/DMT trials in Parkinson’s disease (PD), MCI, and Alzheimer’s disease (AD) reported benefits for PD-related movement disorders and MCI-related cognitive decline, but evidence for AD remains limited, constrained by small samples, variable testing methods, and non-standardized protocols (Wu et al., 2022; Class C). These findings were previously easy to misread as digital-DMT effects; they are not.

### 4.3 Populations with chronic diseases and special health conditions: mind–body integrated digital interventions

Class A evidence for anxiety-focused digital DMT remains thin. Conceptual XR-DMT work describes preventive programmes that integrate wearable biosensing and AI-driven adjustment and that, unlike recreational virtual dance, follow a structured therapeutic frame (mirroring, embodied cognition, rhythm synchronization) (Radanliev, 2025; Class A, conceptual). A randomized trial of digital dance-art therapy in China (Qiu et al., 2025; Class A) found that a three-month, twice-weekly smart-fitness-mirror programme reduced anxiety-related scores relative to traditional studio dance in 48 dancers with none-to-mild anxiety; positive affect improved more clearly than negative affect, which remained mixed. This is direct digital dance-therapy evidence, but it is a small sample of dancers rather than a clinical anxiety-disorder cohort, and PANAS is not a dedicated anxiety diagnostic scale.

Because resting heart rate variability (HRV) of anxious patients is lower than that of healthy individuals (Cohen’s d = 0.61) (Cheng et al., 2022), XR-DMT designs often specify low-intensity (40–50% of maximum heart rate), slow-paced movement and prolonged diaphragmatic breathing as a candidate route to vagal regulation. Conceptual and related XR-DMT papers propose that such programmes can yield anxiety and HRV/EDA changes (Radanliev, 2025; Class A, conceptual); these physiological claims should be read as design hypotheses pending adequately powered trials, not as demonstrated digital-DMT effects. Class C conventional DMT evidence indicates that benefits are not uniform across special clinical populations: DMT was associated with greater physical-tension improvement in eating-disorder patients, whereas art therapy showed relatively greater emotional-tension effects in first-episode psychosis, in a small quasi-experimental sample (Vignapiano et al., 2026).

Therapeutic responses therefore diverge by clinical group and, critically, by evidence class. Conventional DMT (Class C) has been reported to ameliorate PD motor dysfunction and MCI cognitive deterioration, while robust AD evidence is lacking (Wu et al., 2022). Traditional Indian dance training enhanced locomotor proficiency among children with Down syndrome relative to neuromuscular rehabilitation (Raghupathy et al., 2022; Class C), which is neither DMT nor digital. RCT-based reviews report positive psychological well-being effects of DMT in breast-cancer survivors (Fatkulina et al., 2021; Class C; 6 studies, N = 385), yet pooled quality-of-life estimates from two DMT RCTs were non-significant (SMD − 0.09, 95% CI − 0.21 to 0.40, *p* = 0.54) (Abu-Odah et al., 2024; Class C). These cancer findings are conventional DMT/dance evidence. Contrary to high-intensity interval training, which may induce transient immune dysregulation via cortisol-mediated peripheral lymphocyte apoptosis (Al-Rawaf et al., 2023), occupational-stress reasoning in this review draws on a general exercise meta-analysis of 115 trials comparing HIIT with moderate-to-vigorous continuous training (Bi et al., 2026; Class D). That synthesis is not DMT-specific and does not directly assess resting-state autonomic homeostasis; it supplies only a dosing analogue (prefer moderate-to-low continuous work). An eight-month, 48-session dance programme (Tao et al., 2022; Class C) showed reduced fatigue (*p* = 0.024) maintained at 12–20 months; this is conventional dance evidence in youth, not occupational digital DMT. XR platforms equipped with MR/AR/VR displays are discussed as a design option for occupational stress-reduction content (Radanliev, 2024; Class A, conceptual), though ‘precision’ here is a design goal rather than a demonstrated outcome. Digital DMT working models for hotline-based psychological assistance illustrate remote delivery feasibility without physiological outcome data (Xu et al., 2024; Class A, conceptual). Adaptive digital notifications driven by reinforcement learning increased daily steps over 24 weeks in adults with diabetes and depression (Aguilera et al., 2024; Class B; *N* = 168) and offer a methodological precedent for personalization, not a DMT effect. Under sports-adaptation principles, progressive physical and cognitive load, mindfulness, diaphragmatic breathing and body scanning are plausible ingredients of future occupational digital DMT (John et al., 2025), not already-proven digital-DMT outcomes.

Read by evidence class rather than by a single certainty label, high-quality randomized trials of digital DMT are currently few in every group. Adjacent conventional DMT/dance and digital non-DMT evidence is relatively more available for working-age and milder presentations, whereas late-stage cognitive and chronic-disease subgroups show greater response variability, smaller samples and poorer designs. That gradient describes where analogue evidence is thicker; it does not establish a GRADE-style certainty ranking of digital DMT, and it should not be taken to mean that digital DMT has been validated in sub-health working adults or childhood disorders.

### 4.4 Cultural compatibility and localized transformation: theoretical conflicts, practical barriers, and integrated adaptation strategies

In general, the evidence base for digital DMT across demographic groups remains suboptimal, characterized by a lack of rigorously designed randomized controlled trials and by the fact that many frequently cited estimates belong to Class B–D analogues. Although preliminary Class A work confirms therapeutic potential, insufficient direct empirical data hinder unified clinical guidelines. Multiple methodological bottlenecks restrict accumulation of reliable digital-DMT evidence: first, widespread heterogeneity in choreography, frequency, duration and digital platforms; second, non-standardized outcome tools; third, convenience sampling and limited samples, plus inherent blinding limits; fourth, scarce long-term follow-up; fifth, still-preliminary neuroimaging and multimodal-physiology work on mechanisms linking digital physical exercise and psychotherapeutic benefit (Millman et al., 2021; Class D).

Before proceeding, it is useful to clarify what ‘Western’ denotes in this context. Dance movement therapy originated in the United States during the 1940s, pioneered by practitioners such as Marian Chace working within psychiatric hospital settings, and was later formalized through the founding of the American Dance Therapy Association in 1966 (Thomas, 1994; Cruz, 2016). The approach subsequently diffused to the United Kingdom and continental Western Europe from the 1970s and 1980s onward, giving rise to parallel professional bodies such as the Association for Dance Movement Psychotherapy UK (Cruz, 2016; Levy, 2005). It is this US–UK–Western-European lineage, together with the individualist, catharsis-oriented psychotherapeutic assumptions embedded within it, that this review refers to collectively as ‘Western’ or the ‘Western context’ in the discussion that follows.

Digital dance movement therapy originating from the West faces three major systematic implementation obstacles in China’s social and cultural context: mismatched cultural applicability of core embodied cognitive concepts, treatment goal misalignment due to differences in domestic mental health perceptions, and insufficient cultural contextual considerations in digital platform design (Table 2). Western embodied cognition emphasizes outward emotional expression through body movements, which runs counter to the traditional Chinese cultural tendency of implicit emotional regulation and restraint. Meanwhile, local residents generally hold deep-rooted stigma toward mental illnesses and are reluctant to receive explicit psychological intervention. In addition, most existing digital platforms adopt Western dance elements, scenes and interaction modes, failing to integrate traditional Chinese mind–body culture and folk art. All these barriers greatly hinder the popularization and practical effect of imported digital DMT in China.

**Table 2:** Cross-cultural conflicts of digital DMT and China-tailored localization framework matrix.

Adaptation dimension	Core barriers stemming from western paradigms	Chinese localised optimisation pathways
Theoretical layer	Western embodied cognition prioritises overt individual emotional externalisation, conflicting with China’s culturally rooted affective reticence	Integrate traditional Chinese holistic mind–body unity doctrines; incorporate slow-paced breathing and movement philosophies from Tai Chi and Qigong to restructure core therapeutic rationale
Technical layer	Western aesthetic-dominated virtual scenarios and movement repositories incompatible with indigenous somatic conventions; geriatric digital literacy disparities unaccounted for	Build a local choreography algorithm library that integrates Tai Chi and national folk dance; embed cultural landmark landscapes (Forbidden City, Jiangnan classical gardens) in the XR environment; simplify the user interface to improve accessibility
Practical layer	Western-centric individual catharsis-focused goals conflict with collectivistic cultural values and pervasive domestic mental illness stigma	Implement the de-stigmatization health concept to replace clinical psychotherapy labels; develop family-centered and community-based digital dance therapy (DMT) group projects

The basis of Western dance therapy reflects the cognitive theory that emphasizes the expression of personal body emotions, which conflicts with the norms of Chinese cultural tendency to restrain emotional expression, especially in elderly groups who resist open emotional catharsis through body movements. Traditional Chinese medicine’s harmonised physique and spirit doctrine shares intrinsic holistic alignment with embodied cognition yet prioritises internal psychosomatic regulation over externalised emotional release, necessitating theoretical reconfiguration centred on indigenous mind–body unity philosophy (Robison et al., 2004; Xiang and Xiaomei, 2026) (Figure 1).

**Figure 1:**
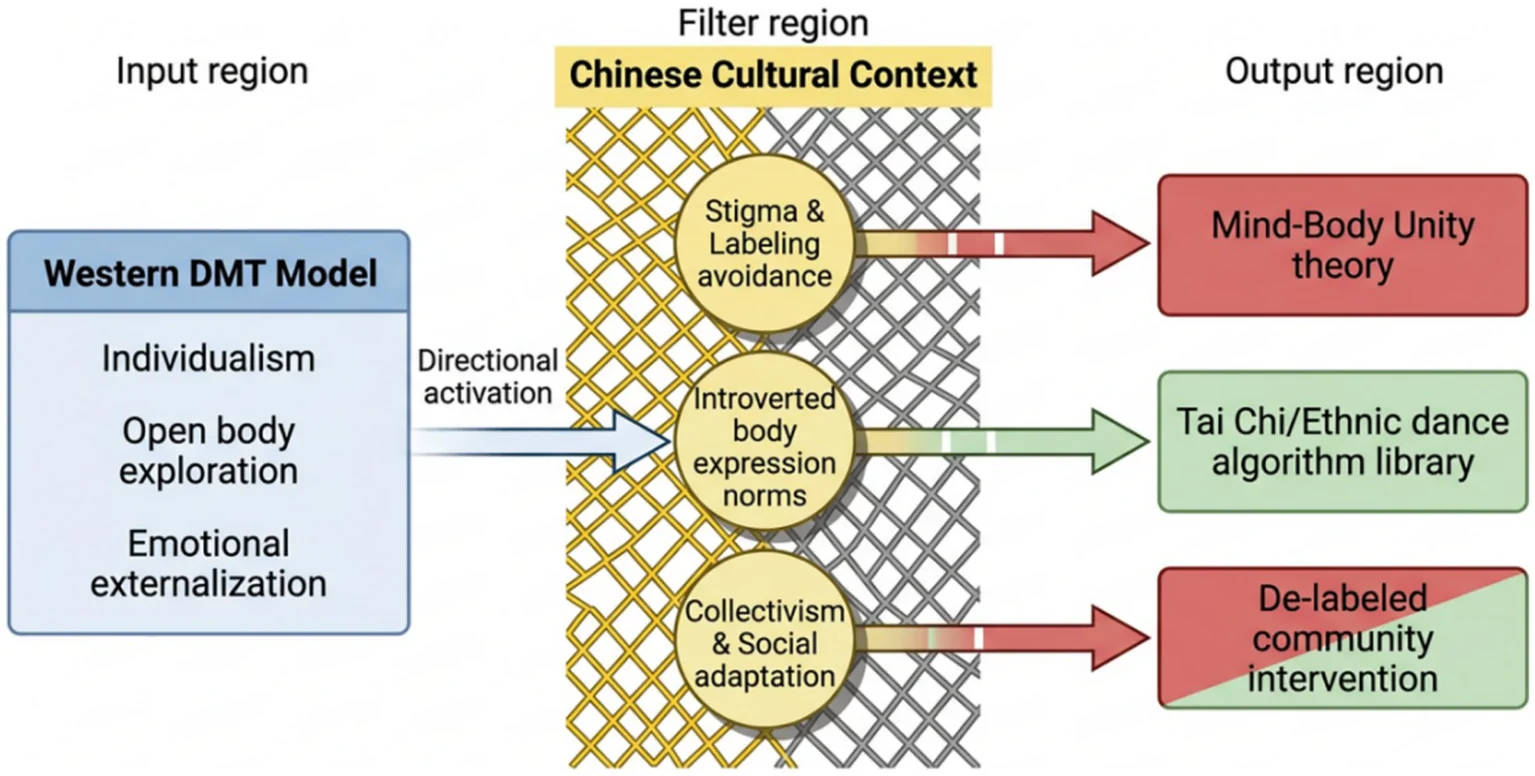
Cross-cultural adaptation conflicts and China-focused localisation remodelling model for digital DMT (author-proposed conceptual model). Input segment denotes conventional Western DMT anchored in individualistic emotional externalisation; intermediate filter represents three Chinese cultural barriers: mental health stigma, normative inhibition of open bodily emotional expression, and collectivistic value orientation; output segment visualises restructured localised solutions: mind–body unity theoretical reframing, indigenous movement algorithm construction, and destigmatised community-centric group intervention.

China’s collectivist cultural milieu shapes three defining domestic mental health characteristics: pervasive psychiatric stigma discourages proactive formal psychological help-seeking (Wang et al., 2021); domestic mental wellness evaluation prioritises social adaptability over hedonic emotional wellbeing (Biao and Xiaoxia, 2024). These patterns should be read as general tendencies rather than uniform traits: attitudes toward emotional expression, help-seeking and therapeutic goals vary considerably by generation, region, urban/rural setting, education, clinical population and individual preference, and younger, more urban cohorts in particular may diverge from the pattern described here. Accordingly, localised DMT reform downregulates cathartic emotional release targets in favour of social adaptation and holistic physical conditioning, adopts destigmatised wellness-centric marketing that substitutes psychotherapy nomenclature, and prioritises family and community group-based intervention design.

## 5 Discussion

Read together, the findings synthesized in Section 4 point to a consistent interpretive pattern. The evidence map in Table 1 shows that Class A digital-DMT sources are few and often conceptual or small-sample, whereas thicker empirical estimates concentrate in Class C conventional DMT/dance and Class B digital non-DMT analogues, and are easier to obtain where implementation is logistically less demanding (working-age and milder presentations) than where unmet clinical need is greatest (advanced neurodegenerative disease, severe psychiatric illness). This is a statement about the distribution of evidence classes, not a study-level GRADE rating. Theoretically, the pattern is consistent with embodied-cognition accounts of therapeutic change: if benefit depends on how reliably a digital system can register and respond to bodily signals, then populations whose movement signals are most atypical or degraded are also those for whom current sensor- and algorithm-based systems are least validated—a mismatch that embodied cognition helps describe but does not yet resolve. Similarly, the cultural incompatibilities identified below are not merely a China-specific implementation footnote; they illustrate that embodied, movement-based interventions encode culturally specific assumptions about selfhood, emotional display and the therapist–client relationship at the level of basic design. Localization therefore cannot be achieved through cosmetic changes such as translating instructions or substituting music, but requires reconsidering the model of embodied selfhood on which the intervention rests. This framework should be understood as a set of testable cultural-adaptation hypotheses rather than an established cultural mapping; confirming which adaptations are acceptable and useful will require participatory co-design with Chinese DMT practitioners, service users, caregivers and community stakeholders, rather than derivation from cultural theory alone.

Existing digital DMT platforms feature systemic cultural design deficits dominated by Western choreography repertoires, non-traditional musical scoring and Eurocentric virtual scenery failing to resonate with Chinese users’ cultural identity and affective preferences (Peng, 2024). Three-tier corrective solutions address this gap: develop proprietary algorithm databases embedding Taijiquan, Qigong and ethnic folk dance paired with traditional Chinese instrumental music (Yue, 2021); integrate canonical domestic cultural landscapes including classical Chinese gardens and imperial architecture within XR immersive environments (Yufei and Jianjun, 2026); simplify graphical interfaces to narrow geriatric digital literacy-derived accessibility gaps to mitigate digital divide inequities.

Over-reliance on technology may weaken the real face-to-face physical and emotional interaction between the therapist and the recipient. The persistent digital divide makes it more difficult for people with poor socioeconomic conditions, older people, or those with low digital literacy to have equal access to services, which may exacerbate existing health inequality; the completion rate of remote interventions is significantly lower among the poor, women, and ethnic minorities (Sayani et al., 2019). Fair digital DMT promotion requires targeted improvement of practitioners’ digital skills, investment in rural digital infrastructure, and provision of a simplified interface architecture accessible to everyone. Large-scale collection of sensitive personal physiological and mental health data through digital platforms will bring huge data governance and privacy risks; contemporary platforms based on generative AI may secretly manipulate behavior through misleading privacy transparency statements, thus changing users’ mode of informed consent disclosure (Wang et al., 2025). The implementation of digital DMT must comply with international data protection regulations similar to GDPR to ensure the confidentiality, integrity, and security of patient data. Clinicians need to conduct a strict risk–benefit assessment of digital aids, clarify the ethical boundaries of remote services, and implement a comprehensive informed consent process before the technical intervention begins. Beyond privacy and access, a clinically oriented digital intervention of this kind also raises safety and governance issues that have not yet been systematically addressed in this review, including cybersickness and other adverse effects of immersive XR use, overexertion and physical injury during unsupervised remote sessions, sensory overload, the risk of inadvertent trauma activation during movement-based emotional release, the absence of clearly defined emergency procedures when a client is in psychological distress during a remote session, sensor failure and model drift over time, algorithmic bias in automated movement assessment, professional licensure and jurisdictional questions for cross-border teletherapy, cybersecurity and secondary use of biometric data, accessibility for people with physical or sensory disability, the cost of implementation for under-resourced providers, and the need for a systematic adverse-event reporting mechanism specific to digital DMT.

Digital transformation requires DMT practitioners to upgrade their multidisciplinary competence framework and combine core treatment expertise with digital literacy, quantitative data interpretation, and remote emotional connection skills. The reform of the training curriculum must integrate digital health ethics, teletherapy practice, and basic data science into formal dance movement therapy (DMT) vocational training to cultivate future interdisciplinary clinicians. As a cutting-edge technology, reinforcement learning offers possibilities for precise and adaptive mental health intervention; biometric data from multi-modal wearable devices can dynamically adjust the choreography intensity, rhythm, background music, and real-time guidance instructions of DMT. Existing randomized trials have verified adaptive digital interventions driven by reinforcement learning, achieving continuous daily steps growth in populations with diabetes and depression within 24 weeks, establishing a methodological precedent for the digital optimization of DMT (Aguilera et al., 2024). At the industry governance level, it is urgent to establish unified technical benchmarks to standardize data privacy standards, the multi-modal biometric ethics guidelines, and evidence-based intervention verification indicators; at the policy level, clinically verified digital DMT schemes should be included in domestic public health resource allocation and medical insurance reimbursement arrangements.

From a broader perspective, the localized development of digital DMT is not merely a technical and clinical improvement of a single intervention method, but also a meaningful exploration of integrating traditional Chinese mind–body wellness culture with modern digital healthcare and global psychotherapy systems. By taking root in local cultural traditions while drawing on advanced international theories and technologies, this field can break the long-standing one-way output of Western psychological intervention models, build a culturally inclusive, independently innovative mental health service system with Chinese characteristics, and provide new ideas and practical references for cross-cultural adaptation of similar digital therapeutic technologies worldwide. Furthermore, as mental health has become an indispensable part of national public health and people’s well-being, the standardized promotion of digital DMT can effectively expand the coverage of grassroots mental health services, realize early screening, intervention and rehabilitation for high-risk groups, and help advance the construction of a comprehensive whole-population health service system. In the long run, the continuous exploration of this interdisciplinary field will also drive the mutual empowerment of sports science, psychotherapy, digital technology and cultural inheritance, and deliver both clinical value and social value for the implementation of the national mental health development strategy.

Beyond its direct relevance to China, this review’s contribution is best understood as a demonstration case for a broader, transferable principle: that the cross-cultural validity of embodied digital mental-health interventions cannot be assumed and must be explicitly theorized and tested wherever such technologies cross cultural boundaries. The specific barriers identified here—affective restraint, psychiatric stigma, and collectivistic rather than individualistic therapeutic goals—are not unique to China. Comparable patterns have been documented across other East Asian societies shaped by Confucian-influenced collectivist norms: independent case studies describe the development of culturally adapted dance/movement therapy programs in Japan (Kaji et al., 2002), indicating that comparable localization efforts have already been undertaken in at least one neighboring East Asian context. A Korea-specific parallel has also been asserted in earlier drafts of this review, but the source previously cited for it (Xu et al., 2024) concerns a working model of DMT for psychological-assistance hotlines rather than an indigenous Korean dance-based program. This lends indirect support to the transferability of the localization framework proposed here—and, plausibly, to Vietnam and to Chinese diaspora communities elsewhere, which share related Confucian-influenced norms even though the specific adaptation pathway may differ by national and linguistic context. More broadly, as digital mental-health platforms increasingly originate in, and are exported from, a small number of high-income Western markets, the analytic approach demonstrated in this review—explicitly separating a technology’s clinical mechanism from the cultural assumptions embedded in its design—offers a transferable method for evaluating cultural fit before large-scale deployment in any Global South or non-Western setting, extending this review’s contribution beyond dance movement therapy to digital mental health technology localization more broadly.

## 6 Conclusion

The in-depth integration of digital technology and dance movement therapy (DMT) is reshaping mental health intervention paradigms. It has evolved from simple technical supplementation into an inclusive, dynamic and precision-driven treatment ecosystem. Virtual reality, artificial intelligence and wearable biosensors have expanded the measurement and delivery options available to DMT. These advances make DMT potentially accessible to populations and clinical settings that previously lacked adequate mental health services. Nevertheless, the field still faces prominent challenges. While preliminary Class A observational and small-trial evidence is encouraging, large-scale, rigorous randomized controlled trials of digital DMT are urgently required to validate long-term effects and underlying neurobiological mechanisms. Adjacent conventional DMT/dance and digital non-DMT findings should not be substituted for those trials. Key constraints also include data security and ethical risks, digital divide inequities, and the redefined roles within human–computer collaborative therapy. Future research must prioritize balancing technological innovation with clinical standards, data application with privacy protection, and technological popularization with equitable service access. The research and standardization of digital DMT call for close interdisciplinary cooperation among clinicians, researchers, neuroscientists, technical developers and bioethicists to establish an evidence-based, ethical and user-centric application framework. Digital tools serve to empower rather than replace therapists. By enhancing diagnostic precision and personalized care under human-centered principles, we can advance the integration, accuracy and inclusiveness of mental health services. Moving forward, sustained efforts should focus on technological innovation and evidence-based validation. Cross-disciplinary teamwork will help balance data utilization and privacy protection, and facilitate the development of localized algorithms, standardized assessment protocols and industrial ethical guidelines. The ultimate goal is to establish a culturally compatible, scalable and practicable digital DMT service system for China, underpinned by humanistic care and technological support, so as to bolster the development of national mental health services.
